# State policies increase vaccination by shaping social norms

**DOI:** 10.1038/s41598-023-48604-5

**Published:** 2023-12-01

**Authors:** Bita Fayaz-Farkhad, Haesung Jung, Christopher Calabrese, Dolores Albarracin

**Affiliations:** 1 University of Pennsylvania, Philadelphia, USA; 2 Texas Tech University, Lubbock, USA; 3 Clemson University, Clemson, USA

**Keywords:** Human behaviour, Health policy

## Abstract

In a survey and four preregistered experiments, we examined if implementing a vaccine-promoting policy is likely to encourage vaccination by shaping the norms of a society. By combining state-level policy data with a longitudinal survey, we found that vaccine-supportive policies and laws are associated with more positive social norms. To establish a causal effect, we conducted four preregistered experiments to gauge the impact of policies, including the government recommendation for children to receive the COVID-19 vaccine and changes in funding for immunization programs. We find that vaccine-supportive policies strengthen the intention to receive an additional recommended COVID-19 booster shot and the intention to vaccinate children against COVID-19. We also find that these effects are mediated by the promotion of social norms supportive of vaccination. In this context, communicating about laws and policies in favor of vaccination may create a culture of vaccination and increase vaccination coverage.

## Introduction

A look at vaccination patterns across the United States suggests stark regional differences. While some areas of the US have achieved full vaccination in more than 80% of their population, other regions still lag behind with rates below 50%^1^. Some of these differences likely stem from disparities in healthcare capacity, modes of production, and vaccine attitudes and beliefs. Other differences are more puzzling. For example, although most states within the Appalachian region struggle with vaccination coverage, West Virginia has been the national leader in vaccination efforts including successful rollout of the COVID-19 vaccine^2^. Given the health and social consequences of vaccination, understanding the roots of these differences is paramount.

Policies as well as norms are likely critical drivers of regional differences in vaccination. Policies involve a variety of regulations and governmental decisions concerning vaccinations, including, for example, state funding support for immunization programs. Social norms are perceptions of the opinions and behaviors of other people^3,4^ including whether the residents of a region have vaccinated and support vaccination. In combination, policies and social norms should form a culture surrounding vaccination, which we define as the practices and norms shared by a group of individuals (for definitions of culture, see^5^).

Understanding regional variations in vaccination is likely to require understanding the relation between policies and social norms. Although policies may reflect social norms^6^, they could also shape social norms by changing individuals’ perception of prevailing behavior^2–6^. As the world prepares for future pandemics, understanding whether vaccine policies actually shape social norms about vaccination is important to decide future regional, national, and global policy actions. Vaccine-promoting policies such as the repeal of vaccination exemptions, recommendations of vaccines by officials, and the amount of funding allocated to immunization programs may strengthen social norms^8,9^.

Two mechanisms have been proposed to explain how changing individuals’ perception of social norms could in turn promote vaccination^7–11^. First is informational impact through which policies may change personal attitudes and beliefs about vaccination, such as the perception that vaccines are beneficial. Second is the normative impact by which policies may elicit conformity, as when people follow the norm to seek approval and/or avoid punishment from other members of their group. In fact, attempts to change behavior through policy implementation and corresponding norms may be most effective when they have both normative and informational impacts.

Most studies examining the relation between policies and social norms have been correlational and found that they are positively related^12–15^. For example, the introduction of lockdown measures in the UK dramatically changed the perception of norms regarding social distancing as measured in a daily survey^13^. However, a critical limitation of any observational study is the difficulty in isolating the effect of the policy on the norm from the reciprocal effect of the norm on the policy^16^. Several attempts were made on this front, with one experimental study identifying a causal direct link between the introduction of social distancing imposed by a central authority and social norms^17^. Likewise, in another study, laws pertaining to sexual activity with minors, selling alcohol to minors, undeclared cash imports into a country, drunk-driving, and speeding have been shown to influence the extent to which people perceive these behaviors as socially appropriate^16^. This study indicates that concerns about social reputation explained some of their findings, suggesting that normative influence is common in this area. However, to the best of our knowledge, no studies have examined the impact of vaccination policies on corresponding social norms.

In this paper, a large panel survey and four controlled pre-registered experiments (one with a nationally representative sample) investigated how vaccination policies shape social norms and consequently affect vaccination. In a probability phone/Internet panel survey, we linked individual respondents to the policies of their state of residence. We then examined the relation between the variation in vaccination policies across states and changes in the social norms reported by survey respondents concerning vaccination. Specifically, we recorded state funding for the vaccination program and the presence of vaccination exemptions in each state. In most states, vaccination exemptions for children can be granted for medical reasons (i.e., the child has some physical ailment that prevents vaccination), religious reasons (e.g., vaccinations violate the parents’ religious beliefs), or philosophical reasons (e.g., vaccinations are not in accordance with the parents’ philosophical beliefs) (for the benefits of exemption repeals, see^17^). Although the United States does not have an influenza vaccination requirement, we hypothesized that state exemption repeals, which have been used to promote vaccination of children, might also shape the norms about vaccination including against influenza.

An important challenge in our observational study is the difficulty in establishing a clear causal direction between policy and social norms as they might influence one another. Four follow-up pre-registered experiments therefore established causality by manipulating information about policies and testing their influence on perceived social norms and vaccination intentions to ensure that policies, rather than other confounding factors, are responsible for the changes in norms and intentions. Given that states cannot be randomized to different policies, we used scenarios about the policies implemented in a city. Specifically, in Study 2, participants were asked to imagine moving to a new city whose government recommended healthy children to vaccinate against COVID-19 or recommended against healthy children vaccinating against the disease. In Studies 3, 4, and 5, participants were asked to imagine moving to a new city in which either the city increased or decreased the funding allocated to its immunization program. In Studies 4 and 5, we also included a control condition in which participants read that the city simply maintained their previous level of funding allocated to the immunization program. Lastly, in Study 5, which was conducted with a nationally representative sample, we assessed whether the influence of policies on behavior was normative or informational by examining the perceived obligation to vaccinate and perceived benefits of vaccination as two mechanisms that can explain shift in behavior. All studies considered the mediating role of social norms on the effect of policies on vaccination intentions. Furthermore, all studies measured a vaccine-unrelated policy to verify that the effects were localized on vaccination and thus addressing a possible experimental demand.

This research makes several critical contributions to the literature. First, we investigate how vaccination policies causally influence vaccination norms. To the best of our knowledge, no study has examined such an influence on the scope of vaccination decisions. Second, we contribute by simultaneously studying the normative and informational processes of the impact of policies on norms. Third, we establish the robustness of the phenomenon by considering three different vaccination policies, including the repeal of vaccination exemptions, recommendations of vaccines by officials, and the amount of funding allocated to immunization programs. All research was carried out in accordance with relevant guidelines and regulations. All experimental protocols were approved by the Institutional Review Board of the University of Pennsylvania, and all participants provided informed consent.

## Results

### Study 1

Study 1 (see Methods) recruited a probability phone/Internet sample of American adults (N = 3005) to participate in a panel survey at four different timepoints across the 2018–2019 influenza season. One of our key dependent variables was social norms, which was measured with two items: (a) How important, if at all, do you think it is that most people in your community get the flu vaccine? (1 = not important at all, 2 = not too important, 3 = somewhat important, 4 = very important) and (b) Think about the people important to you. How likely, if at all, are they to want you to get the flu vaccine this flu season? (1 = not likely at all, 2 = not too likely, 3 = somewhat likely, 4 = very likely). Both items were asked at all four timepoints. We created a composite score of social norms for each participant by averaging their responses to the two items and then calculated the state-level score of social norms by obtaining the mode of individuals’ composite score within each state. We used modes rather than means as the mode is the majority opinion in the state. In comparison, the mean could be averaging over several disparate groups which does not capture the majority opinion. Because participants reported their state of residency, the state level policies including the percentage of state funding allocated to the state’s immunization program and the state’s repeal of vaccine exemptions for non-medical reasons were linked to participants’ norm score based on their state of residency. Participants’ intentions to vaccinate against influenza were measured at every timepoint as long as participants did not report receiving the vaccine using the item: How likely, if at all, are you to get the flu vaccine before or during this flu season? (1 = not likely at all, 2 = not too likely, 3 = somewhat likely, 4 = very likely). Finally, participants answered demographic questions about their age, education, household income, trust in government, as well as political ideology. These variables were introduced for control purposes.

We used structural equation modeling to examine the effects of policies on social norms and their mediating role in the path from policies to intentions with several controls. First, we controlled for individual age, education, political ideology, and household income as these factors often correlate with vaccination. Second, we also controlled for participants’ trust in government as this factor could impact responsiveness to government-imposed vaccination policies. Third, regions with less positive vaccination norms may also have lower vaccination access^18^, lower level of funding allocated to the immunization program, and more vaccination exemptions^18,19^. Therefore, we controlled for the state-level number of primary care providers per capita, the state rate of residents without health insurance coverage, and the rate of residents without a regular source of health care^19^. All continuous predictors were z-standardized before the analyses. As presented in Table 1, the analyses showed that higher levels of state funding allocated to the immunization program and the repeal of vaccination exemptions permitted for non-medical reasons were positively associated with social norms, which were then positively associated with stronger intentions to receive the influenza vaccine (*b*_1_ = 0.014, *SE* = 0.005, *z* = 2.86, *p* = 0.004; *b*_2_ = 0.027, *SE* = 0.009, *z* = 2.93, *p* = 0.003). However, policies and norms can co-evolve. Specifically, rather than policies causing norms, both state policies and social norms could be related to the perceived threat of influenza and therefore influence vaccination intentions. Hence, to establish the direction of causality, we conducted four experiments.

**Table 1:** Effects of state policies on norms and intentions.

	*B*	*se*	*z*	*p*	95% CI	
**Path to norms **						
Repeal of vaccine exemptions	0.169	0.031	5.41	0	0.108	0.230
Percentage of funding allocated to the immunization program	0.312	0.052	5.99	0	0.210	0.414
Ideology	− 0.007	0.008	− 0.88	0.376	− 0.022	0.008
Education	− 0.002	0.008	− 0.26	0.795	− 0.019	0.014
Income	0.021	0.008	2.48	0.013	0.004	0.037
Age	− 0.002	0.008	− 0.21	0.83	− 0.018	0.014
State uninsured rate	0.101	0.015	6.53	0	0.071	0.131
State rate of individuals without usual source of care	− 0.095	0.015	− 6.27	0	− 0.124	− 0.065
State number of primary care physicians per capita	− 0.165	0.048	− 3.45	0.001	− 0.258	− 0.071
Time point 2	2.063	0.025	83.88	0	2.014	2.111
Time point 3	0.596	0.028	21.65	0	0.542	0.650
Time point 4	0.407	0.029	13.83	0	0.349	0.465
**Path to intentions**						
Norms	0.085	0.025	3.37	0.001	0.036	0.135
Repeal of vaccine exemptions	0.022	0.055	0.41	0.681	− 0.084	0.129
Percentage of funding allocated to the immunization program	0.198	0.091	2.17	0.03	0.020	0.376
Ideology	− 0.015	0.014	− 1.13	0.257	− 0.042	0.011
Education	0.073	0.015	5.03	0	0.045	0.102
Income	0.029	0.014	1.98	0.047	0.000	0.057
Age	0.107	0.014	7.48	0	0.079	0.135
State uninsured rate	− 0.016	0.027	− 0.58	0.563	− 0.069	0.037
State rate of individuals without usual source of care	− 0.063	0.026	− 2.38	0.017	− 0.114	− 0.011
State number of primary care physicians per capita	− 0.232	0.083	− 2.79	0.005	− 0.395	− 0.069
Time point 2	− 0.377	0.050	− 7.49	0	− 0.476	− 0.278
Time point 3	− 0.677	0.057	− 11.89	0	− 0.789	− 0.566
Time point 4	− 0.806	0.067	− 11.95	0	− 0.938	− 0.674

*B*: standardized estimates. *Se*: standard errors. *CI*: Confidence intervals.

### Study 2

We next conducted a preregistered experiment (https://aspredicted.org/ja3bz.pdf) to establish the causal impact of policies on social norms and to test whether norms mediate the effects of policies on vaccination intentions. Specifically, Study 2 examined the impact of recommending or not recommending COVID-19 vaccination for healthy children on perceived social norms and vaccination intentions. We conducted this study in March 2022 when the Surgeon General of Florida newly announced the state’s plan to formally recommend against COVID-19 vaccination for healthy children. Thus, it is unlikely that participants had already formed strong attitudes toward the recommendation we tested at the time of the study.

Study 2 (see Methods) recruited 200 (N for analysis = 190) individuals from Prolific (www.prolific.com) to participate in an online health survey. The study used a one-way between-subjects design with conditions recommending in favor or recommending against COVID-19 vaccination of healthy children. Participants were randomly presented with one of the following two scenarios about moving to a different city for a new job: (a) that the city implemented a policy that recommends healthy children to vaccinate against COVID-19 or (b) that the city implemented a policy that recommends against healthy children to get a COVID-19 vaccine. In both scenarios, participants also read about the implementation of another policy that prohibits the use of plastic bags in grocery stores to prevent them from guessing the main hypothesis and thus reducing bias in their responses. The results concerning plastic bags also served as a control policy, as our manipulation should only influence answers about vaccination. After reading about the scenarios, participants indicated their intentions to vaccinate their children against COVID-19 and to use plastic bags, and their perceived social norms defined as the extent to which participants believed others in the city vaccinate their children against COVID-19 and use plastic bags. Participants then responded to demographics and manipulation checks. The results of the manipulation checks, which suggested that our experimental procedures worked as expected, appear in the Methods section.

**Figure 1:**
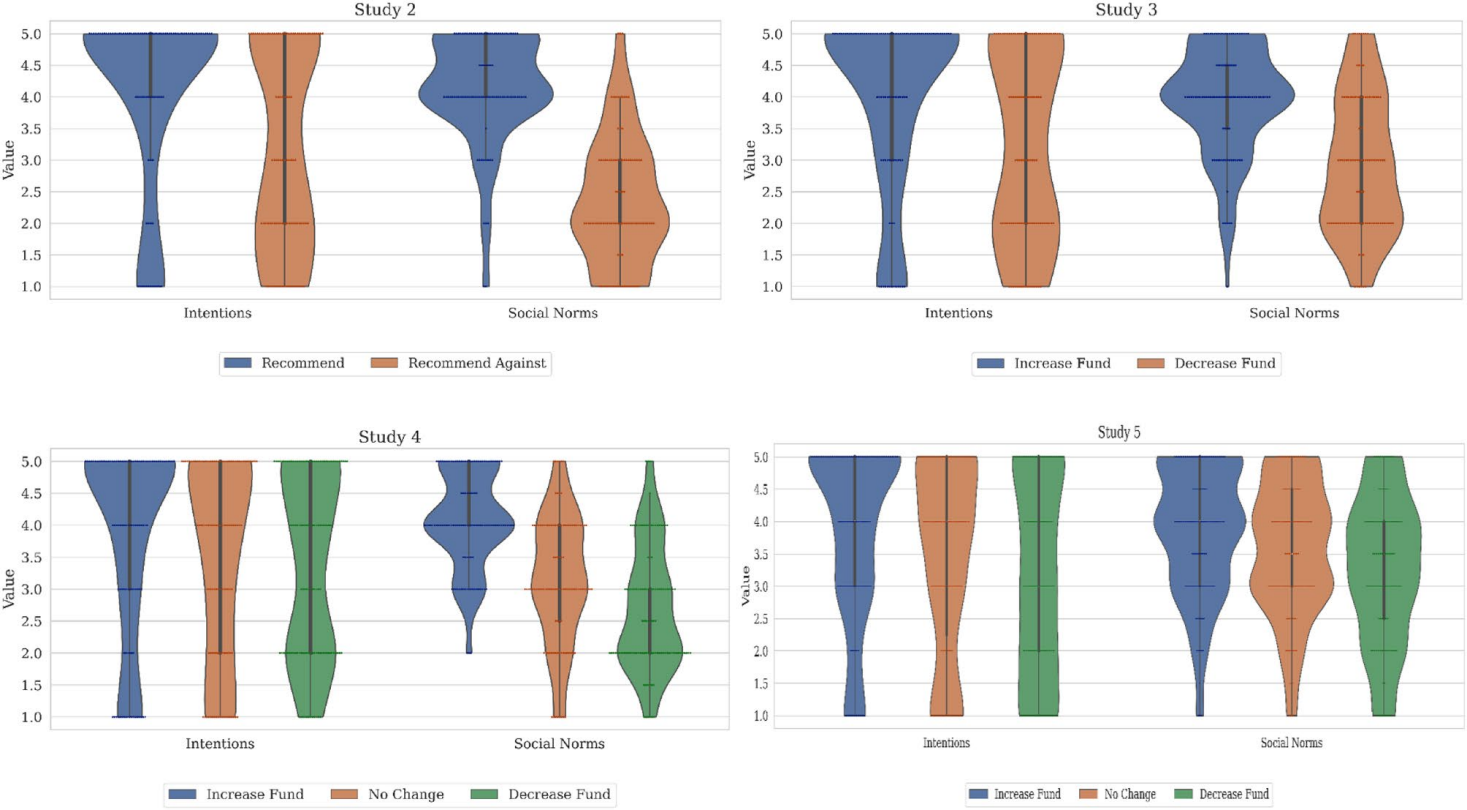
Effects of policy on intentions to vaccinate and their perceived social norms.

Analyses of variance with policy as a between-subjects factor showed that different policies significantly influenced both the intentions to vaccinate children against COVID-19, *F*(1, 188) = 18.12, *p* < 0.001, and social norms about vaccination, *F*(1, 188) = 171.81, *p* < 0.001. These results appear in Fig. 1. Specifically, participants in the pro-vaccination policy condition reported stronger intentions to vaccinate (Recommend vaccination: *M* = 4.13, *SD* = 1.42) than did participants in the anti-vaccination policy condition (Recommend against vaccination: *M* = 3.19, *SD* = 1.60). Likewise, participants in the pro-vaccination policy condition reported higher perceived social norms (Recommend vaccination: *M* = 4.15, *SD* = 0.87) than did participants in the anti-vaccination policy condition (Recommend against vaccination: *M* = 2.34, *SD* = 1.02). Thus, these results conceptually replicated the findings from our panel survey with an experimental methodology. As intended, the policy manipulation did not affect either participants’ intentions to use plastic bags (Recommend vaccination: *M* = 1.56, *SD* = 1.09 and Recommend against vaccination: *M* = 1.59, *SD* = 1.12, *F*(1, 188) = 0.03, *p* = 0.87), or their social norms about the use of plastic bags (Recommend vaccination: *M* = 4.34, *SD* = 0.89 and Recommend against vaccination: *M* = 4.20, *SD* = 0.97, *F*(1, 188) = 1.05, *p* = 0.31). The experimental findings on both norms and intentions remained when we restricted the sample to adult parents of children (Recommend vaccination: social norms *M* = 4.18, *SD* = 0.95; intentions *M* = 2.58, *SD* = 1.68; Recommend against vaccination: social norms *M* = 1.71, *SD* = 1.01; intentions *M* = 3.6, *SD* = 1.67). However, due to the lack of sufficient power in the subsample, the effects on intentions to vaccination against COVID-19 were only marginally significant (effect on social norms *F*(1, 37) = 62.04, *p* < 0.001; effects on intentions *F*(1, 37) = 3.56, *p* = 0.067).

The validity of an experiment may be threatened when participants guess the study hypothesis and simply follow the perceived experimental demand. If that were the case, our results could stem from participants believing that policies shape norms and simply responding in ways that confirm such belief. Thus, we explored participants’ thoughts about the relation between policy and norms by asking them to rate four statements on a scale from 1 (strongly disagree) to 5 (strongly agree). These statements included (a) The new policies were implemented because residents supported/demanded these policies in the first place, (b) When the city implements a new policy, the policy persuades the residents to follow the city recommendation, (c) New policies are unlikely to be implemented without initial support from residents, and (d) New policies have the ability to change people’s opinions and behavior. We tested the robustness of the findings by removing those who had stronger beliefs in items a and c than items b and d and found similar effects on both intentions, *F*(1, 88) = 9.18, *p* = 0.003, and social norms about vaccination, *F*(1, 88) = 54.92, *p* < 0.001. As the results were preserved, we concluded that the experimental findings were present even without participants having a clear naïve theory about policies affecting norms. Our subsequent experiments led to the same conclusion, which led to the decision to report these particular results for the other experiments only in the supplement.

**Figure 2:**
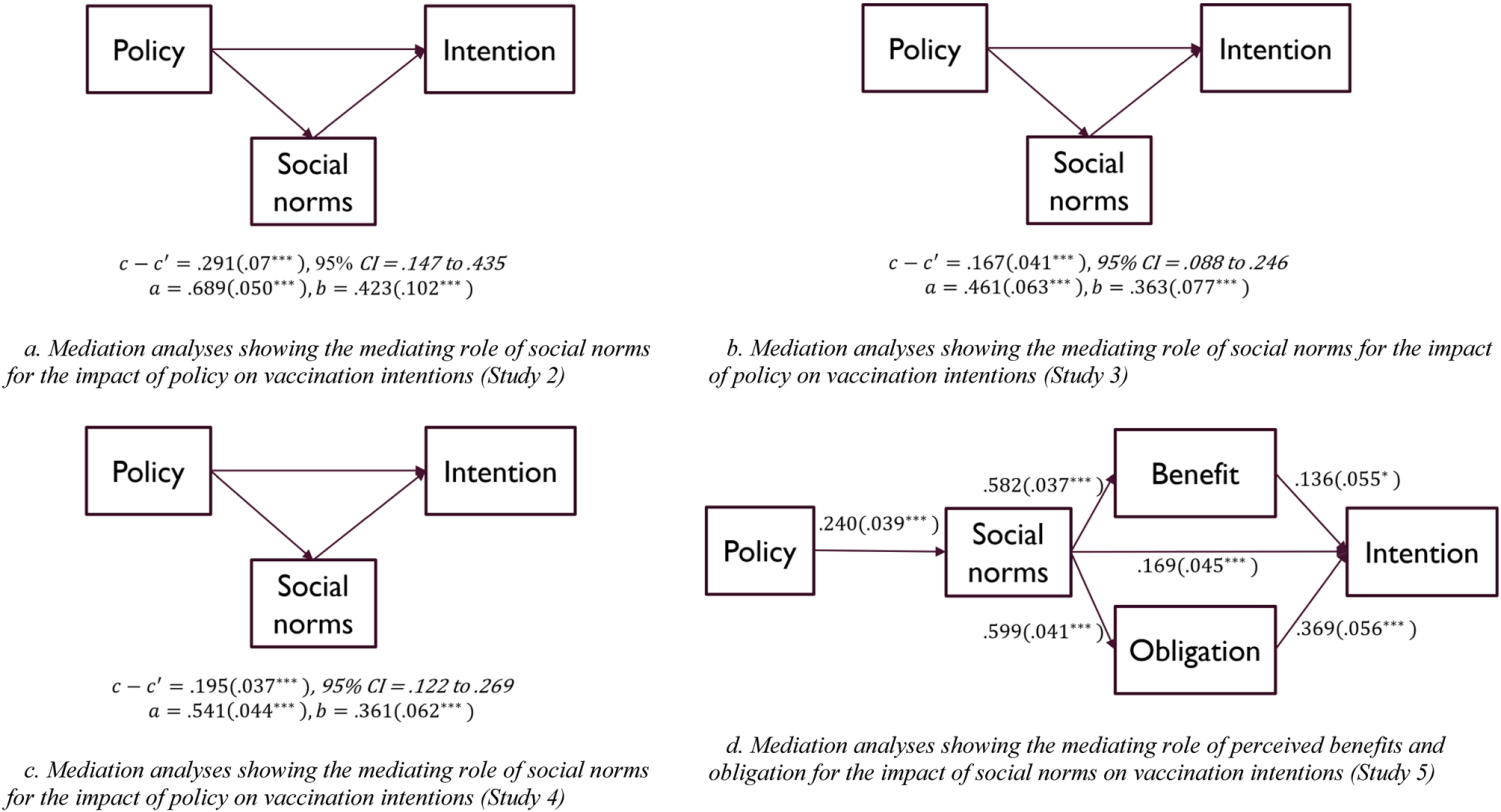
Mediation models.

We then examined whether social norms significantly mediated the impact of the policy on intentions to vaccinate children against COVID-19. To establish whether there was an indirect effect, bootstrapping was applied, calculating the confidence intervals with the bias-corrected percentile method^18^. The results from a bias-corrected mediation analysis conducted with 2000 bootstrapping samples appear in Fig. 2a. As predicted in our preregistration, we found a significant indirect effect whereby recommending the vaccine was associated with stronger social norms, which then led to higher intentions to vaccinate children. Thus, recommending healthy children to vaccinate against COVID-19 produced higher intentions to vaccinate children by exerting mediating effects on social norms, *indirect effect* = 0.29, *SE* = 0.07, *z* = 3.96, 95% CI = 0.15–0.45 (Fig. 2a). Importantly, the direct effect of recommending vaccination against COVID-19 on intentions to vaccinate children was not statistically significant, *b* = 0.005, *SE* = 0.1, *z* = 0.04, 95% CI = − 0.20 to 0.21, implying that social norms fully mediated the impact of the policy on intentions.

The findings from our preregistered Study 2 supported the conclusions from Study 1. To conceptually replicate and test the robustness of the effects observed in our previous studies, we conducted another three preregistered experiments that examined the effects of funding allocated to the immunization program on social norms and vaccination intentions.

### Study 3

Study 3 was a preregistered experiment (https://aspredicted.org/qe7ye.pdf) that recruited 200 individuals via the online platform Prolific to participate in an online health survey (see Methods). The study involved an experimental design with policy as a between-subjects factor. Participants were asked to imagine themselves moving to a different city for a new job, and that the city implemented a new policy that consisted of: (a) increasing the percentage of city funding allocated to the immunization program or (b) decreasing the percentage of city funding allocated to the immunization program. As in Study 2, all participants also read about the implementation of a policy that prohibits the use of plastic bags in grocery stores, which served as a control. After reading the scenarios, participants indicated their intentions to receive an additional recommended COVID-19 booster shot and use plastic bags, as well as their perceived social norms measured as the extent to which they believed others in the city vaccinate and use plastic bags. Finally, participants responded to manipulation checks, whose results are described in the Methods section.

The findings from our preregistered Study 3, which supported the conclusions from the earlier studies, appear in Fig. 1. As preregistered, there was a significant effect of the manipulated policy on intentions to receive an additional COVID-19 booster shot, *F*(1, 198) = 14.58, *p* < 0.001, and social norms about getting a booster, *F*(1, 198) = 54.75, *p* < 0.001. Specifically, participants in the increased funding condition reported stronger intentions to vaccinate (increased funding: *M* = 4.01, *SD* = 1.42) than did participants in the decreased funding condition (decreased funding: *M* = 3.22, *SD* = 1.51). Likewise, participants in the increased funding condition reported higher perceived social norms (increased funding: *M* = 3.95, *SD* = 0.85) than did participants in the decreased funding condition (decreased funding: *M* = 2.92, *SD* = 1.11). As before, the policy manipulation did not affect intentions to use plastic bags (increased funding: *M* = 1.56, *SD* = 1.09 and decreased funding: *M* = 1.59, *SD* = 1.12, *F*(1, 198) = 1.40, *p* = 0.24) nor social norms about the use of plastic bags (increased funding: *M* = 1.56, *SD* = 1.09 and decreased funding: *M* = 1.59, *SD* = 1.12, *F*(1, 198) = 0.76, *p* = 0.39).

We next examined whether perceived social norms mediated the impact of the city-level funding policy on intentions to receive an additional COVID-19 booster shot through a bias-corrected mediation analysis with 2000 bootstrap samples. As in Study 2, we found a significant indirect effect where increased city-level funding for the immunization program was associated with more positive perceived social norms, which was in turn associated with stronger intentions to receive an additional COVID-19 booster shot. Thus, increased city-level funding for the immunization program strengthened intentions to receive an additional COVID-19 booster shot by increases in social norms, *indirect effect* = 0.17, *SE* = 0.04, *z* = 4.12, 95% CI = 0.09–0.25 (Fig. 2b). As before, the direct effect of city funding for the immunization program on intentions to receive an additional COVID-19 booster shot was not statistically significant, *b* = 0.09, *SE* = 0.08, *z* = 1.21, 95% CI = − 0.06 to 0.24, suggesting that norms fully mediated the impact of the policy on vaccination intentions. We next conducted another preregistered experiment that replicated Study 3 with the addition of a control condition in which there was no change in the immunization program’s funding policy.

### Study 4

We conducted Study 4, a preregistered experiment (https://aspredicted.org/vy2sf.pdf) with a sample recruited via the online platform Prolific (*N* = 361; *N* for analysis = 352) (see Methods). In this study, participants were randomly assigned to one of three experimental conditions of funding allocated to the immunization program in a city to which they were about to move: (a) increased, (b) no change, and (c) decreased funding allocation. In all three scenarios, participants also read about the implementation of a ban on the use of plastic bags in grocery stores in the city. As in the prior studies, after reading about the scenarios, participants indicated their intentions to receive an additional recommended COVID-19 booster shot and use plastic bags as well as their perceived social norms for each behavior. As in Studies 2 and 3, participants then responded to manipulation checks whose results are reported in the Methods.

The results from Study 4 appear in Fig. 1 and confirmed to our preregistered hypothesis. As before, an analysis of variance of intentions to receive a booster as a function of the manipulated policy as a between-subjects factor showed a significant effect on vaccination intentions, omnibus *F*(2, 349) = 4.53, *p* = 0.01. In particular, intentions to receive an additional COVID-19 booster shot were descriptively stronger in the increased funding condition (increased funding: *M* = 3.91, *SD* = 1.47) than either the decreased funding condition (decreased funding: *M* = 3.37, *SD* = 1.44) or the control funding condition (no change: *M* = 3.48, *SD* = 1.51). Intentions were significantly stronger in the increased funding condition than the decreased funding condition (contrast = 0.54, *SE* = 0.19, *p* = 0.01). The no change in funding (control) condition fell in the middle although it did not differ significantly from either of the other conditions. As in the earlier studies, the effects of the policy manipulation were not significant for intentions to use plastic bags (increased funding: *M* = 1.41, *SD* = 1.01, no change: *M* = 1.4, *SD* = 0.96, and decreased funding: *M* = 1.31, *SD* = 0.86, *F*(2, 349) = 0.52, *p* = 0.59).

We also analyzed the effects of the policy on social norms and found a significant omnibus effect, *F*(2, 349) = 75.28, *p* < 0.001. Social norms were stronger in the increased funding condition (increased funding: *M* = 4.09, *SD* = 0.57) than either the decreased funding condition (decreased funding: *M* = 2.69, *SD* = 0.97; contrast = 1.41, *SE* = 0.12, *p* < 0.001) or the stable funding condition (no change: *M* = 3.14, *SD* = 1.01; contrast = 0.96, *SE* = 0.12, p < 0.001).

As with intentions, the policy had no effects on social norms surrounding the use of plastic bags (increased funding: *M* = 4.40, *SD* = 0.72, no change: *M* = 4.28, *SD* = 0.9, and decreased funding: *M* = 4.44, *SD* = 0.79), *F*(2, 349) = 1.21, *p* = 0.30.

We next examined whether perceived social norms mediated the impact of the policy on intentions to get an additional COVID-19 booster shot. A bias-corrected mediation analysis using 2000 bootstrapped samples revealed a significant indirect effect whereby increased funding (vs. decreased or no change) was associated with more positive perceived social norms, which were in turn associated with stronger intentions to receive the COVID-19 booster. Thus, increasing funding for the immunization program heightened intentions through a mediating increase in social norms, *indirect effect* = 0.20, *SE* = 0.04, *t* = 5.23, 95% CI = 0.122–0.269 (Fig. 2c). Moreover, the direct effect of the policy on intentions was not significant, *b* =  − 0.04, *SE* = 0.06, *z* = − 0.71, *95% CI* = − 0.164 to 0.076, implying that the impact of the policy was entirely mediated by social norms.

In conclusion, the results from the first four studies supported the prediction that implementing a new policy supportive of vaccination can promote vaccination by producing positive norms around vaccination. Next, we conducted a final preregistered experiment to assess whether vaccination policies influence perceived benefits of vaccination and/or obligations to vaccinate as a way of eliciting vaccination. Whereas perceived benefits reflect informational influence, perceived obligation reflects normative influence.

### Study

We conducted Study 5, a preregistered experiment (https://aspredicted.org/fu5n6.pdf) (see Methods) with a nationally census-based representative sample recruited via the online platform Dynata (www.dynata.com) (N = 633). In this study, participants were randomly assigned to one of three conditions identical to Study 4. Study procedures were identical to Study 4 with the exception that we measured two possible mediators of the policy: (a) perceived benefits of vaccination and (b) perceived obligation to vaccinate (see the *Methods* section to review how these indexes are constructed).

Intentions as well as norms were examined using analysis of variance. As preregistered, intentions, *F*(2, 632) = 13.31, *p* < 0.001, and social norms, *F*(2, 630) = 18.91, *p* < 0.001, were a function of the manipulated policy. These results appear in Fig. 1 and revealed that all differences across the conditions were significant (contrasts > 0.3, *p* < 0.01 in all cases). The bias-corrected mediation analysis with 2,000 bootstrapped samples revealed a significant indirect effect whereby the experimental condition was associated with more positive perceived social norms, which were in turn associated with stronger intentions to receive the COVID-19 booster. Thus, increasing funding for the immunization program strengthened intentions to get the COVID-19 vaccine by increasing pro-vaccination social norms, *b* = 0.11, *SE* = 0.02, *t* = 5.68, *p* < 0.001. Unlike past studies, the direct effect of increasing funding for the immunization program on intentions to receive an additional COVID-19 booster shot remained significant albeit smaller, *b* = 0.09, *SE* = 0.04, *t* = 2.4, *p* = 0.02, implying that norms partially mediated the effect of the policy on intentions. The indirect effect observed represented 55.71% of the total effect.

We also analyzed the effects of the policy manipulation on perceived benefits of vaccination and perceived obligation to vaccinate, which were significant, *F*(2, 619) = 4.29 for perceived benefits and *F*(2, 619) = 6.84 for perceived obligation; *p* < 0.05 in each case. We next analyzed whether perceived benefits and/or obligation mediated the effects of norms on intentions by introducing the two mediators simultaneously (Fig. 2d). Our mediation analysis based on 2,000 simulated resamplings revealed a significant indirect effect whereby more positive perceived social norms due to the policy manipulation were associated with higher perceived obligation and benefits, which were then associated with stronger intentions to receive the COVID-19 vaccine (total effect for the two mediators: *indirect effect* = 0.30, *SE* = 0.03, *z* = 10.01, 95% CI = 0.241 to 0.359). Furthermore, the direct effect of social norms on intentions to receive an additional COVID-19 booster shot remained significant, *b* = 0.17, *SE* = 0.05, *z* = 3.7, 95% CI = 0.08–0.259, implying that the effect was only partially mediated by these mechanisms. Still, the indirect effect observed represented 63.97% of the total effect, implying a medium effect on the basis of Cohen’s guidelines^19^. Thus, the results suggested that vaccination policies shape behavioral intentions through both informational and normative influences.

## Conclusion

According to the theory^2–6^, implementing a new law or policy may not only directly channel people to perform the desired behavior but also promote the desired behavior by shaping the norms of society. By combining state-level policy data with a panel survey study, we found that the policies designed to promote vaccination were positively correlated with social norms and vaccination intentions. To better establish a causal effect, we conducted four preregistered experiments, one with a nationally representative sample, examining the impact of policies, such as recommending for or against healthy children to vaccinate against COVID-19 and increasing or decreasing funding for the immunization program. In all four experimental studies, the presence of vaccine-supportive policies increased the intention to receive a vaccine by promoting social norms supportive of vaccination. The mediating role of norms in the impact of policies on behavior was greater than the direct effect of the policies, even though the direct effect represents the standard mechanism that underlies the use of policies to influence behavior. Two mechanisms (i.e., informational, normative) can be used to understand why policies shape behavior through shaping norms^7–11^. Study 5 demonstrated that policies exerted both informational and normative influences to promote vaccination outcomes.

It is not clear how much our results might generalize to other policies or vaccines. Our study focused on policies that ease a behavior without necessarily rewarding it, such as recommending vaccination or increasing vaccination funding. Polices that pair negative behavior with punishments (e.g., restricting one’s ability to work or travel when one chooses not to vaccinate) may not have similar effects on norms due to their potential for psychological reactance. However, a recent study found that the social distancing measures introduced during the pandemic in the UK had a stronger impact on social norms than government announcements about the dangers of social interactions^17^. Likewise, it is possible that the effect of policies might be less effective for more established vaccines such as MMR, as people might have already formed strong attitudes about the vaccine and thus their vaccination decisions would be less susceptible to policy effects.

Another potential limitation of this study is the use of hypothetical scenarios to investigate the causal impact of policies on norms. One could test the hypotheses of our study using different methods. For example, future research may use natural experiments to examine the role of vaccination policies. In this respect, it is reassuring that our main results are in line with those of other recent studies on other health topics, using different methodologies and data^14,17^.

Despite these caveats, our findings inform public policy by showing that, in addition to implementing policies in favor of vaccination, increasing public awareness about these policies will be key. Actively communicating about pro-vaccination policies, such as using various channels to promote and increase awareness of the implemented policies, therefore represents a major lever for public policy to increase vaccination coverage in the United States and possibly in other areas of the world.

## Materials and methods

All research was carried out in accordance with relevant guidelines and regulations. All experimental protocols were approved by the Institutional Review Board of the University of Pennsylvania, and all participants provided informed consent.

### Study 1

#### Participants and design

We analyzed four timepoints from a national probability panel study assessing beliefs and behavior concerning vaccines. The study included a probability-based, nationally representative sample of American adults who were randomly selected from the AmeriSpeak panels of the National Opinion Research Center (NORC) at the University of Chicago between September 2018 and October 2019 and who provided responses at four different timepoints (time 1 = September 2018, time 2 = November 2018, time 3 = January 2019, time 4 = March 2019). This sampling frame covered 97% of U.S. households via a two-stage stratified sampling^20^. Respondents primarily completed the survey online (*N* = 2289), although a small percentage answered the survey over the phone (landlines and cell phones) to avoid a bias favoring people who use the Internet. The total sample included 3005 participants.

Participants were 47% male and 53% female. With respect to race, the starting sample was 60% non-Hispanic white, 14% Black, 15% Hispanic, 6% Asian, and 5% other. The mean age was 48.4 years old (*SD* = 18), with a median education level of an associate degree and a median annual household income level of $50,000–$59,999. The distributions of age, sex, race, and income showed trivial differences from the general US population. However, there was a discrepancy in education, with more of our sample having a college degree than the 2018 U.S. Census estimates. The discrepancy was due to lower completion rates among lower education respondents in September. To mitigate this problem, these respondents were successfully targeted for retention in follow-up timepoints with more incentives and reminders.

### Measures

#### Norms

The study included two items with a 4-point scale from 1 to 4: (a) How important, if at all, do you think it is that most people in your community get the flu vaccine? (1 = not important at all, 2 = not too important, 3 = somewhat important, 4 = very important) and (b) Think about the people important to you. How likely, if at all, are they to want you to get the flu vaccine this influenza season? (1 = not likely at all, 2 = not too likely, 3 = somewhat likely, 4 = very likely). We averaged the two responses (r = 0.42) at each timepoint for each person and calculated a state-level norm by obtaining the mode within each state. We used modes rather than means because the mode is the majority opinion in the state. In comparison, the mean could be averaging over several disparate groups without actually reflecting any singular one.

#### Intentions

Intentions were assessed through a one-item question, How likely, if at all, are you to get the flu vaccine before or during this flu season? This item was rated on a 4-point scale (1 = not likely at all, 2 = not too likely, 3 = somewhat likely, 4 = very likely). Intentions were measured at every timepoint as long as participants did not report receiving the vaccine.

#### Demographics

Participants self-reported their age and biological sex. Annual household income was measured on a 21-point semi-linear scale, with the first 15 points increasing in $5000 increments from < $15, followed by $10,000 increments until the final point at > $250,000. Because the distribution was normal around the mean of $55,000 (SD = $20,000), we treated this variable as continuous in all analyses.

#### State-level policy data

The vaccine policies of the repeal of vaccination exemptions and percentage of state funds allocated to the immunization program and additional state-level items that could potentially covary with state policies (i.e., the state-level number of primary care providers per capita; the state rate of residents without health insurance coverage; and the rate of residents without a usual source of care) to influence vaccination and social norms were also linked to the sample. See^21^ to review the state-level data used in the analysis with corresponding sources and year of data used.

### Study 2

#### Participants and design

In March 2022, we recruited 200 participants from Prolific for a 2-cell, between-subjects experimental design. 10 participants did not complete the entire survey which left us with a final sample size of 190. Participants were asked to imagine that they have moved to a new city for a new job and that the city has implemented new policies. This information appeared within a broader description of the city, the complete text of which appears in the Supplementary Materials. Respondents were randomly assigned to read one of the following two policy scenarios: (a) “the city implemented a policy that recommends healthy children to vaccinate against COVID-19 to help protect against COVID-19” (Recommendation in favor condition) or (b) “the city implemented a policy that recommends against healthy children getting a COVID-19 vaccine” (Recommendation against condition). In both scenarios, participants also read about implementing a second policy “the city implemented a policy that prohibits the use of plastic bags in grocery stores.”

Participants were 49% male and 48% female and were 36.97 (*SD* = 14.77) years old on average. With respect to race, 67% of the respondents were white, 3% were Black, 10% were Asian, and 13% reported being from other racial groups. With respect to ethnicity, 7% were Hispanic. Furthermore, participants had a median education level of a bachelor’s degree and a median annual household income level of $50,000–$59,999.

#### Measures

One dependent measure was the intention to vaccinate children against COVID-19, which was measured on a 1–5 scale in response to the question, As a resident in the city, how likely are you to vaccinate your kid against COVID-19 if you had a child? (1 = extremely unlikely, 2 = somewhat unlikely, 3 = neither unlikely or likely, 4 = somewhat likely, 5 = extremely likely). Another dependent measure was social norms, which asked participants, What are your thoughts about what other residents in the city you just moved to would generally do? Participants answered the question by marking their agreements with two statements: Most residents would vaccinate their child against COVID-19, and The majority of residents will probably vaccinate their children against COVID-19 from 1 to 5 (1 = strongly disagree, 2 = somewhat disagree, 3 = neither agree or disagree, 4 = somewhat agree, 5 = strongly agree).

#### Manipulation checks

We administered manipulation checks by asking participants about the policy implementation scenario and whether they were assigned to recommendation in favor of vaccination or recommendation against vaccination. As expected, most participants (85% of participants in the recommendation scenario and 89% of participants in the recommendation against scenario) correctly specified the condition to which they were assigned. We therefore concluded that our manipulation was successful.

#### Probes for demand

We explored participants’ thoughts by asking them to rate 4 statements about the relation between policy and norms on a scale from 1 (strongly disagree) to 5 (strongly agree). These statements included (a) The new policies were implemented because residents supported/demanded these policies in the first place, (b) When the city implements a new policy, the policy persuades the residents to follow the city recommendation, (c) New policies are unlikely to be implemented without initial support from residents, and (d) New policies have the ability to change people’s opinions and behavior. We found that policies significantly influenced both the intentions to vaccinate children against COVID-19 [*F*(1, 98) = 8.8, *p* = 0.004] and social norms about vaccination [*F*(1, 98) = 127.05, *p* < 0.001] among those who had stronger beliefs in items a and c than items b and d. Likewise, we found that policies significantly influenced both the intentions to vaccinate children against COVID-19 and social norms about vaccination [*F*(1, 88) = 54.92, *p* < 0.001] among those who did not have stronger beliefs in items a and c than items b and d [*F*(1, 88) = 9.18, *p* = 0.003].

### Study 3

#### Participants and design

In March 2022, we recruited 200 participants from Prolific who participated in a 2-cell, between-subjects experimental design. Using scenarios similar to the earlier ones, participants were told that they had moved to a new city for a new job and that the city implemented new policies (a) “the city implemented a policy that allocates more funding toward a program to increase immunization rates among residents” (increase in funding condition), (b) “the economic hardship has led the city to reduce funding on their immunization program” (decrease in funding condition). In both scenarios, participants also read about the city implementing a ban on plastic bags, as in Study 2.

Participants were 49% male and 47% female and were 39.39 (*SD* = 15.68) years old on average. With respect to race, 65% of the respondents were white, 10% were Black, 10% were Asian, and 6% were other races. With respect to ethnicity, 6% were Hispanic. Furthermore, participants had a median education level of an associate degree and a median annual household income level of $50,000–$59,999.

#### Measures

One dependent measure was the intention to vaccinate, which was measured by the question, As a resident in the city, how likely are you to get an additional recommended COVID-19 booster shot? (1 = extremely unlikely, 2 = somewhat unlikely, 3 = neither unlikely or likely, 4 = somewhat likely, 5 = extremely likely). The second dependent measure was social norms, which was measured with the questions, What are your thoughts about what other residents in the city you just moved to would generally do? The items of the scale are Most residents would receive an additional recommended COVID-19 booster shot, and The majority of residents will probably receive an additional recommended COVID-19 booster shot with responses measured on a scale from 1 to 5 (1 = strongly disagree, 2 = somewhat disagree, 3 = neither agree or disagree, 4 = somewhat agree, 5 = strongly agree).

#### Manipulation checks

We checked our manipulation by asking participants about the policy implementation scenario and whether they were assigned to increase in funding or decrease in funding scenario. As expected, most participants (98% of participants in the increase in funding scenario and 93% of participants in the decrease in funding scenario) correctly specified the condition to which they were assigned. We therefore concluded that our manipulation was successful.

#### Probes for demand

These measures were the same as in experiment 2. As expected, we found that policies significantly influenced both the intentions to vaccinate against COVID-19, *F*(1, 113) = 6.89, *p* = 0.009, and social norms about vaccination, *F*(1, 113) = 35.93 *p* < 0.001 among those who had stronger beliefs in a and c than items b and d. Likewise, we found that policies significantly influenced both the intentions to vaccinate against COVID-19, *F*(1, 83) = 5.67, *p* = 0.02, and social norms about vaccination, *F*(1, 83) = 15.92, *p* < 0.001 among those who did not have stronger beliefs in a and c than items b and d.

### Study 4

Participants and Design. In April 2022, we recruited 361 participants (*N* for analysis = 352) recruited from Prolific and entailed a 3-cell, between-subjects experimental design. Participants were told that they moved to a new city for a new job and that the city has implemented new policies (a) “the city implemented a policy that allocates more funding toward a program to increase immunization rates among residents.” (increase in funding condition), (b) “the economic hardship has led the city to reduce funding on their immunization program” (decrease in funding condition), and (c) “there is no change in state funding on their immunization program” (control condition). All participants also read about the plastic bag ban as in prior studies.

Participants were 50% male and 47% female and were 37.78 (*SD* = 14.17) years old on average. With respect to race, 69% of the respondents were white, 5% were Black, 11% were Asian, and 15% were other races. With respect to ethnicity, 10% were Hispanic. Furthermore, participants had a median education level of a bachelor’s degree and a median annual household income level of $40,000–$49,999.

#### Measures

Intentions and social norms were measured with the items used in Study 3.

#### Manipulation checks

These measures were the same as in the prior experiments. As before, most participants (97% of participants in the increase in funding scenario, 94% of participants in the control condition, and 97% of participants in the decrease in funding condition) correctly specified the condition to which they were assigned. We therefore concluded that our manipulation was successful.

#### Probes for demand

We explored participants’ thoughts by asking them the items used in studies 2 and 3. As before, we found that policies significantly influenced social norms about vaccination against COVID-19 among those who had stronger beliefs in a and c than items b and d, *F*(2, 196) = 53.22, *p* < 0.001. Likewise, we found that policies significantly influenced social norms about vaccination against COVID-19 among those who did not have stronger beliefs in a and c than items b and d, *F*(2, 150) = 22.27, *p* < 0.001. However, due to the lack of sufficient power in both subsamples, the effects on intentions to vaccination against COVID-19 were not statistically significant *F*(2, 196) = 2.8, *p* = 0.063; *F*(2, 150) = 2.26, *p* = 0.108.

### Study 5

#### Participants and design

In May 2022, we recruited a nationally representative sample of 633 participants from Dynata. Similar to Study 4, this study entailed a 3-cell, between-subjects experimental design. Participants were told that they have moved to a new city for a new job and the city has implemented one of the following three policies: (a) “the city implemented a policy that allocates more funding toward a program to increase immunization rates among residents” (increase in funding condition), (b) “the economic hardship has led the city to reduce funding on their immunization program” (decrease in funding condition), and (c) “there is no change in state funding on their immunization program” (control condition).

Participants were 45.99 (*SD* = 49.61) years old on average. With respect to race, 61% of the respondents were white, 12% were Black, 5% were Asian, and 3% reported other races. With respect to ethnicity, 12% were Hispanic. Furthermore, participants had a median education level of an associate degree and a median annual household income level of $40,000–$49,999.

*Measures*. Intentions and social norms were measured with the items used in Studies 3 and 4. In addition, this study measured two potential mediators. Depending on their assigned condition, participants were told. When the city implements a policy that allocates more funding toward a program to increase immunization rates among residents/When the economic hardship has led the city to reduce funding for their immunization program/When there is no change in state funding on their immunization program, what are your thoughts? They then rated 16 statements on a scale from 1 (strongly disagree) to 5 (strongly agree). These statements were used to create two indexes. An index of perceived benefits was created by averaging the items: (a) Other people getting this vaccine tells me the vaccine is safe, (b) Other people getting this vaccine tells me the vaccine is necessary, (c) Other people getting this vaccine tells me the vaccine protects me from infection, (d) The city's funding policy tells me the vaccine is safe, (e) The city's funding policy tells me the vaccine is necessary, and (f) The city's funding policy tells me the vaccine protects me from infection, (Cronbach’s alpha = 0.85). An index of conformity was created by averaging the items: (a) Other residents will judge me negatively if I don't get this vaccine, (b) I feel pressured to get this vaccine, (c) The government may punish me if I don't get this vaccine, (d) The government will judge me negatively if I don't get this vaccine, (e) I think getting this vaccine is the right thing to do, (e) I think I should get this vaccine, (f) I think getting this vaccine is a moral thing to do, (g) I think getting this vaccine is a responsible thing to do, (h) I think other residents expect me to get this vaccine, and (i) Other residents think it's important that I get this vaccine (Cronbach’s alpha = 0.83).

#### Manipulation checks

These measures were the same as in the prior experiments. As in Study 4, most participants (55% of participants in the increase in funding scenario, 51% of participants in the control condition, and 71% of participants in the decrease in funding condition) correctly specified the condition to which they were assigned. We therefore concluded that our manipulation was successful.

### Supplementary Information

Supplementary Information.

## Data Availability

The datasets generated and/or analyzed during the current study are available in the OSF repository https://osf.io/v84ah/?view_only=1643babae04d447992b4c10f187a679e.
