# Supplementary material for: State policies increase vaccination by shaping social norms
Source: Sci Rep. 2023 Dec 1;13:21227. doi: 10.1038/s41598-023-48604-5 (PMC10692068; doi:10.1038/s41598-023-48604-5)
Supplement: Supplementary file 1 — Supplementary Information. [file 41598_2023_48604_MOESM1_ESM.pdf]

Supplementary Materials

Study 2 (Recommendation vaccine condition)

Imagine that you have moved to a different city for a new job. While living in the city will be a new experience, you are excited to learn more about your community.

Figure A 1. Study material (Recommendation vaccine condition)

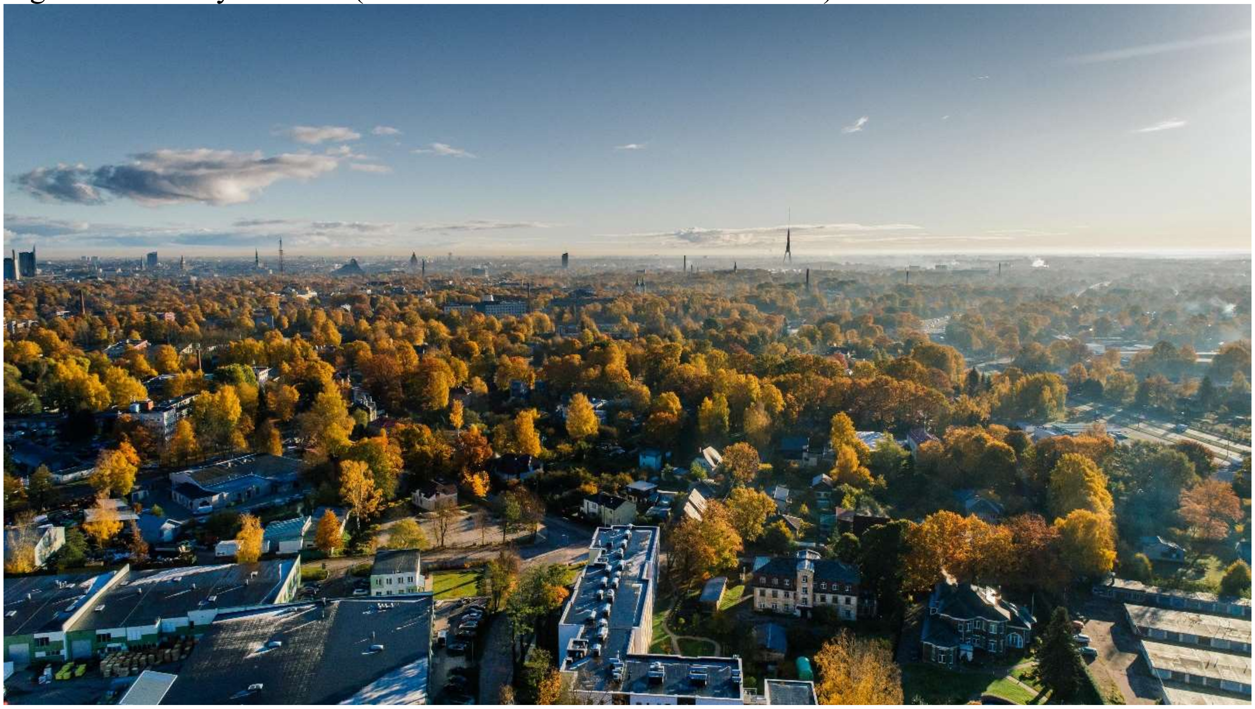

Source: www.pexels.com.

The city has an estimated population of 125,000 residents. 65% identify as White, 10% identify as Hispanic or Latino, 15% identify as Asian, 8% identify as Black or African American, and 2% identify as other or mixed race. About 88% of the residents ages 25 and older have a high school diploma. The city has several new recreational parks and shopping centers. There are also community events that happen all year, as well as a weekly farmer's market.

The city has recently implemented two new policies. First, the city implemented a policy that **prohibits the use of plastic bags in grocery stores**. Second, the city implemented a policy that **recommends healthy children to vaccinate against COVID-19** to help protect against COVID-19**.**

Study 2 (Recommendation against vaccine condition)

Imagine that you have moved to a different city for a new job. While living in the city will be a new experience, you are excited to learn more about your community.

Figure A 2. Study material (Recommendation against vaccine condition)

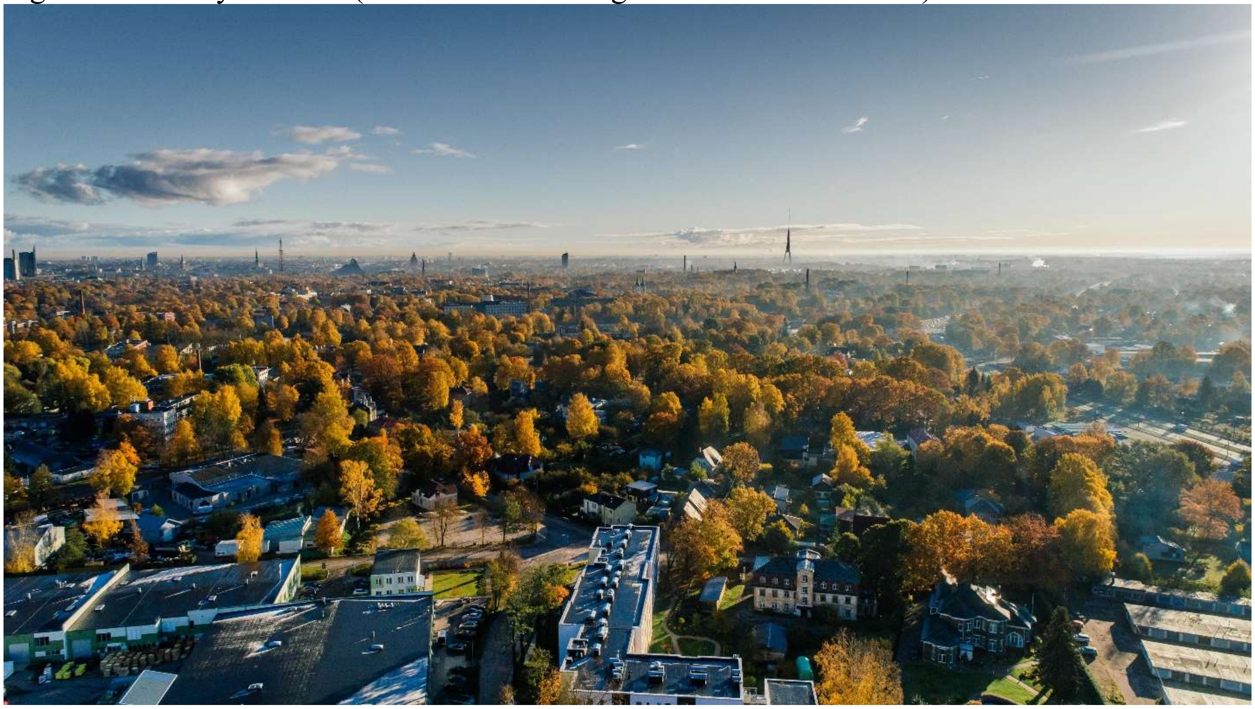

Source: www.pexels.com.

The city has an estimated population of 125,000 residents. 65% identify as White, 10% identify as Hispanic or Latino, 15% identify as Asian, 8% identify as Black or African American, and 2% identify as other or mixed race. About 88% of the residents ages 25 and older have a high school diploma. The city has several new recreational parks and shopping centers. There are also community events that happen all year, as well as a weekly farmer’s market.

The city has recently implemented two new policies. First, the city implemented a policy that **prohibits the use of plastic bags in grocery stores**. Second, the city implemented a policy that **recommends against healthy children getting a COVID-19 vaccine.**

Study 3 (Increase funding condition)

Imagine that you have moved to a different city for a new job. While living in the city will be a new experience, you are excited to learn more about your community.

Figure A 3. Study material (Increase funding condition)

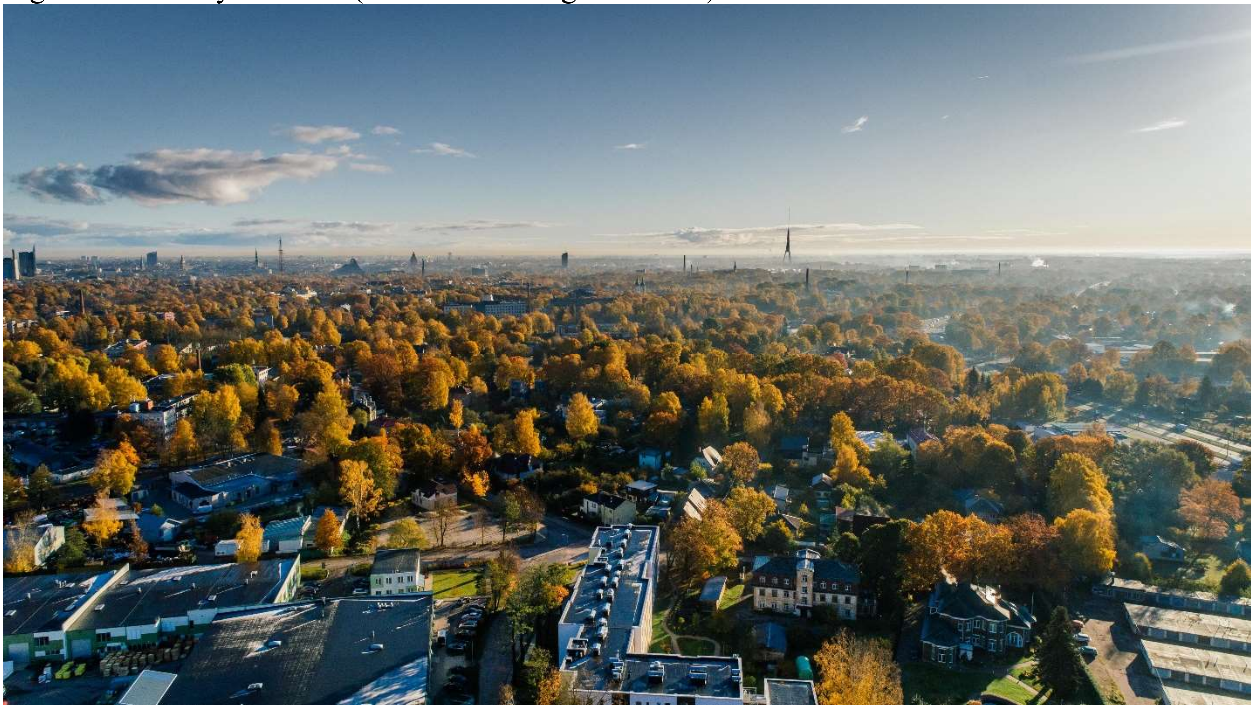

Source: www.pexels.com.

The city has an estimated population of 125,000 residents. 65% identify as White, 10% identify as Hispanic or Latino, 15% identify as Asian, 8% identify as Black or African American, and 2% identify as other or mixed race. About 88% of the residents ages 25 and older have a high school diploma. The city has several new recreational parks and shopping centers. There are also community events that happen all year, as well as a weekly farmer's market.

The city has recently implemented two new policies. First, the city implemented a policy that **prohibits the use of plastic bags in grocery stores**. Second, the city implemented a policy that **allocates more funding toward a program to increase immunization rates among residents**.

Study 3 (Decrease funding condition)

Imagine that you have moved to a different city for a new job. While living in the city will be a new experience, you are excited to learn more about your community.

Figure A 4. Study material (Decrease funding condition)

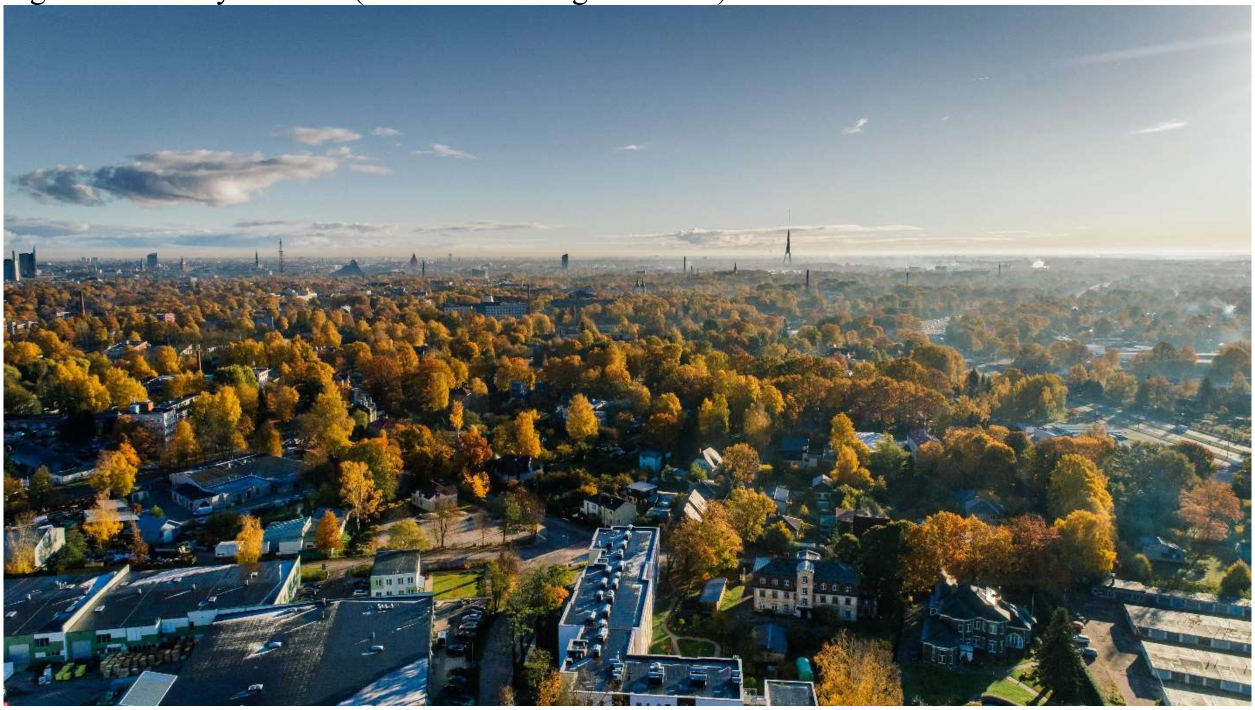

Source: www.pexels.com.

The city has an estimated population of 125,000 residents. 65% identify as White, 10% identify as Hispanic or Latino, 15% identify as Asian, 8% identify as Black or African American, and 2% identify as other or mixed race. About 88% of the residents ages 25 and older have a high school diploma. The city has several new recreational parks and shopping centers. There are also community events that happen all year, as well as a weekly farmer's market.

The city has recently implemented two new policies. First, the city implemented a policy that **prohibits the use of plastic bags in grocery stores**. Second, the economic hardship has led the city to **reduce funding on their immunization program**.
